# The Relationship between Workplace Violence and Innovative Work Behavior: The Mediating Roles of Employee Wellbeing

**DOI:** 10.3390/healthcare8030332

**Published:** 2020-09-10

**Authors:** Xiang Zhou, Samma Faiz Rasool, Dawei Ma

**Affiliations:** 1 School of Management, Guangzhou University, Guangzhou 510006, China; zx@gzhu.edu.cn (X.Z.); madavid@gzhu.edu.cn (D.M.); 2 School of Innovation and Entrepreneurship, Entrepreneurship Institute, Guangzhou University, Guangzhou 510006, China; 3 Postdoctoral Station of Statistical, Guangzhou University, Guangzhou 510006, China

**Keywords:** workplace violence, employee wellbeing, innovative work behavior

## Abstract

It has been contended that violence is prevalent in the workplace, and there has been increasing research interest into its potential effects. Human interactions at workplaces are apparent. However, the interactions among humans may have positive or negative dimensions. Usually, the positive or negative interactions between workers lead to different outcomes. Sometimes, they lead to a productive working environment; however, in some cases, they lead to toxicity among workers. In this study, we investigate the impact of workplace violence (WV) on innovative work behavior (IWB). Specifically, it examines the impact of the three dimensions of WV, namely, harassment, mobbing, and sabotage. Moreover, employees’ wellbeing mediates the relationship between WV (harassment, mobbing, and sabotage) and IWB. A questionnaire survey approach was used in this study. The target population were the workers of SMEs entrepreneurs located in Guangdong Province (China). The results confirm that, in the direct relationship, WV (harassment, mobbing, and sabotage) has a negative relationship with innovative IWB. Moreover, results also confirm that employee wellbeing is mediated between WV (harassment, mobbing, and sabotage) and IWB. Therefore, the empirical results of this paper identify that workplace violence reduces employees’ innovative work behavior by reducing their subjective and eudemonic wellbeing, which further broadens the perspective of IWB’s motivation analysis. Practical implications for small and medium enterprise organizations have also been discussed in this paper.

## 1. Introduction

China has experienced dramatic industrialization, urbanization, and economic growth in the last three decades. Small- and medium-sized enterprises (SMEs) play a crucial role in the Chinese economy, providing about 80% of jobs in urban China [1]. Compared to large enterprises, SMEs tend to be less regulated in safe work environment. In addition, employees in SMEs usually have low wages and a high level of workplace violence [2]. Rural-to-urban migrants consist of the majority of the employees in SMEs who migrate from less developed areas to more developed areas in China [3]. It was estimated that the number of migrant workers had reached 263 million (19.4% of the total population) in 2012 [4]. Compared to local residents, workplace violence problems and suicides are more likely to occur in these migrants [1]. Studies have shown that workplace violence of workers in SMEs is worse than both the general population and those in large enterprises [5,6]. However, workplace violence in SMEs is increasing day by day, which is affecting the innovate work behavior (IWB) of the workers.

The ever-increasing environmental uncertainty and dynamism in the 21st century prompt organizations to increasingly rely on innovation to maintain or improve competitiveness and efficiency [7,8]. Particularly, employees’ innovative work behavior (IWB), which refers to a set of activities that create new products and optimize workflows by developing, adopting, and applying new ideas, has become a key asset for companies to adapt to a dynamic business environment and maintain a competitive advantage. [9]. Therefore, attention to the personal and situational factors that influence IWB of organizational behavior researchers is growing [10]. Among them, varieties of personal factors are of interest, such as social/group context and individual differences [11]. Meanwhile, some of the environmental factors, such as organization culture and climate [12] relationship with their supervisors [8] and job characteristics [13], are also investigated.

In terms of organizational culture and climate, the impact of workplace violence is of great concern to organizations. Workplace violence is cruel and violent treatment of persons or danger that notes a risk to the health and safety of employees [14] As a factor of workplace climate, workplace violence is widespread, but due to shyness, difficulty in obtaining evidence and fear of retaliation, etc., few victims of workplace violence are willing to come forward and talk about these experiences [15]. The avoidance and silence of the victims make this phenomenon challenging to notice, which makes it less of a concern for scholars [16]. However, it is precisely the invisibility of the violence that makes victims suffer, so employees who suffer from workplace violence often lack wellbeing. Employee wellbeing refers to a series of happiness and joyful emotions subjectively generated by human beings based on their own security and satisfaction [17]. According to Maslow’s hierarchy of needs theory, security is the most basic happiness need of human beings, while insecurity is not mentioned for other higher level needs [18]. Workplace violence is a climate factor that destroys victims’ sense of security, so its impact on their wellbeing is undoubted.

**Figure 1:**
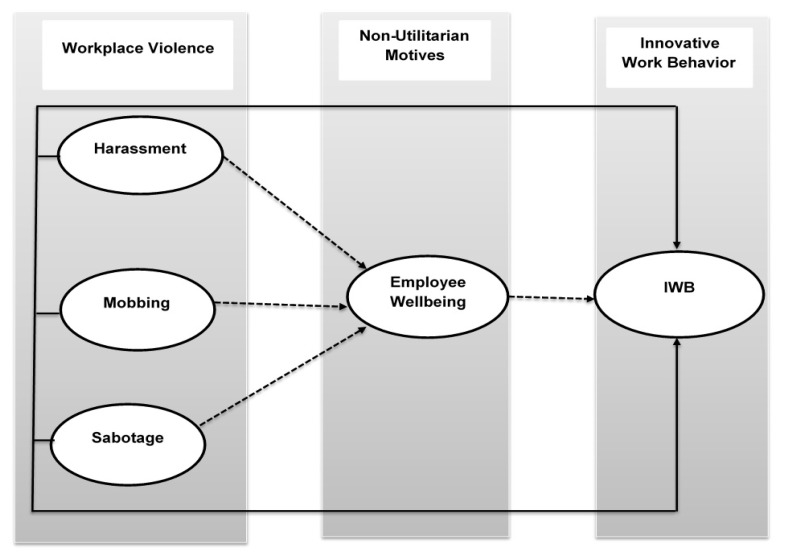
Proposed research model.

Although numerous studies have explored the psychological processes that promote innovative work behavior [8,19,20] these studies have not, to date, clearly defined the characteristics of organizational environments that contribute to the cognitive processes, which is innovation-supportive, nor have they fully evaluated the role of these organizational environments and cognitive processes in promoting individual innovation [8,21]. In order to further explore the influencing factors of IWB on the basis of these research gaps, we propose an empirical model which holds that workplace violence (i.e., harassment, mobbing, and sabotage) harms the IWB of employees through individual emotional processes (i.e., employee wellbeing) (Figure 1 depicts this model).

Several potential theoretical contributions are included in this study. First, by assessing the relationship between the three dimensions of workplace violence (i.e., harassment, mobbing, and sabotage) and innovative work behaviour, from a negative perspective, this finding explores the environmental factors that are not conducive to innovation in organizations, making up for the shortcomings of previous studies that focus on positive environmental factors but ignore negative environmental factors. Second, for the first time, we attempt to explore employees’ innovative behaviours on the basis of conservation of resources (COR) theory and eliminate previous IWB studies on the basis of expectation theory, which paid significant attention to the psychological process of employees’ subjective expectations and ignored the deficiencies of the psychological process affected by objective environmental factors [22]. Finally, we examine the mediating effects of employee wellbeing on workplace violence and innovative work behaviour, and this finding suggests that IWB is not an absolute utilitarian behaviour but may also be an unconscious organizational citizenship activity providing useful implications for the design of nonphysical incentives for innovation (e.g., nonviolent organizational climate). Therefore, on the basis of the above explanation, the research questions (RQ) shown below are developed.

**RQ1:** How WV influences the innovative work behaviour.

**RQ2:** How does employee wellbeing intervene between WV and innovative work behaviour?

This paper consists of five parts. The first part reviews the literature about workplace violence, innovative work behavior, and employees’ wellbeing and develops the hypotheses of this study. The second section entails details about the research methods of this research. In the third section, analysis and results are explained in detail. The fourth section includes a discussion and the implications of the study, and the fifth and last section describes the conclusion, limitations and future research direction.

## 2. Literature Review

### 2.1. Workplace Violence

Workplace violence is any act or threat of physical violence, harassment, intimidation, or other threatening disruptive behavior that occurs at the worksite. It ranges from threats and verbal abuse to physical assaults and even homicide. It can affect and involve employees, clients, customers, and visitors. The term “workplace violence” has multiple factors that include: workplace harassment, workplace bullying workplace mobbing, workplace incivility, workplace sabotage, workplace stalking, and others. Rasool, Wang [14] introduced the three dimensions that define workplace violence, such as harassment, mobbing, and sabotage. Moreover, Ferris, Lian [23] present in their study that WV has three dimensions: harassment, mobbing, ostracism, and sabotage. These dimensions are defined as follows: workplace harassment is humiliation and terrorization of one individual by another in the workplace [24]; workplace mobbing means bullying of an individual by a group in any context, such as by family or peers, at school or work, in the neighborhood, community, or online [25]; workplace sabotage is the generic term for a whole host of tricks, deviltry, and assorted nastiness that can remind the boss how much he needs his workers [26].

### 2.2. Employee Well-Being

Employee well-being relates to all aspects of working life, from the quality and safety of the physical environment, to how workers feel about their work, their working environment, the climate at work and work organization [27,28]. Daniels, Gedikli [29] demonstrate that employee well-being comprises both physical and mental aspects. The mental aspects of employee well-being include apprehension, illness, depression, fatigue, self-respect, and anxiety, whereas physical aspects include headache, and muscular discomfort. Employee well-being is important for the success of the organization [30]. Poor sense of well-being will result in increased health insurance costs and lower productivity. Therefore, it is necessary for companies to understand how their programs impact an employee’s well-being [31].

### 2.3. Innovative Work Behaviour

Innovative work behavior refers to a series of behaviors of employees that give full play to their creativity to think and optimize their work procedure and performance on the basis of routine work. Generally, this series of behaviors include identifying problems in work, creatively introducing new and useful ideas, implementing relevant ideas in a targeted way, and so on. Innovative work behavior differs from the creativity of employees who focus on discovering and generating ideas [32], and creativity is the process of initiating new and useful ideas [33]. In addition to the generation of new ideas, IWB includes numerous activities to adopt, implement, develop, and modify new ideas around performance improvement [34]. Compared with creativity, IWB is more purposeful than creativity and covers the process of implementation and optimization of new ideas.

Therefore, creativity can be regarded as a dimension of IWB, because it is part of the first stage of IWB, in which workers identify the performance gaps and creatively propose ideas for improvement [35]. Innovative work behavior has also been found to be broader than pro-activeness constructs, such as proactive work behavior [36] and personal initiative [37], which focus on individuals’ inclination to implement ideas proactively. Pro-activeness constructs concentrate on the tendency of individuals to actively implement ideas, but they cannot capture the part of ideas generated in the process of innovation [38]. Therefore, IWB is a concept covering the generation and purposeful implementation of new ideas, which has important theoretical and practical values for explaining the user experience, workflow, and product design optimization of enterprises. Under the guidance of expectation theory, existing research on IWB mainly discusses the driving mechanism of innovation output expectation on individual IWB [21]. Previous studies on IWB have attached great importance to the driving effect of positive internal factors on individual IWB, while less attention has been paid to the binding effect of external negative factors on IWB [39].

Although more and more scholars have begun to explore this field in recent years, the core of relevant research has been the impact of leaders’ characteristics on employees’ IWB [8,38]. The impact of a collective environment composed of leaders and other employees on specific employees’ IWB remains unexplored [8,12,40].

## 3. Hypothesis Development

### 3.1. Workplace Violence and IWB from the Perspective of COR Theory

The intra- and inter-relationships of employees at the workplace show a clear image of the workplace environment [41,42]. To guide our theoretical arguments about the interactive effect of the workplace environment and IWB, we draw from COR theory, which argues that the motivation of employee behavior is to protect and maintain the resources they have. [43].

Recently, OB scholars have been increasingly paying attention to COR theory and it is used to investigate the relationship between workplace environment and employee behaviors [44,45]. According to the definition, IWB is that employees must invest extra time and energy in generating behaviors in addition to routine tasks. According to COR theory, the pressure of resource loss or potential loss will urge employees to try to protect existing resources and reduce investment to avoid further risks [44]. From this perspective, workplace violence, as a negative working environment, will affect employees’ IWB from at least two aspects. First, workplace violence causes employees to spend more time and energy on unproductive matters, such as maintaining interpersonal relationships [14]. People’s time and energy are limited. When a part of their time and energy has to be used to deal with non-productive affairs, employees will inevitably face the dilemma of reducing production efficiency or maintaining necessary work efficiency through extra overtime. This can lead to a sense of loss where the employees’ efforts are not rewarded; that is, the reduction in IWB due to the actual loss of resources. Second, workplace violence will reduce employees’ expectations of the resources they can obtain in specific jobs in the future [45], including opportunities for promotion, income improvement and job satisfaction. Therefore, employees will also reduce their efforts in this position due to their low expectation of future resource acquisition, thus affecting the production of IWB.

To further clarify the internal mechanism of workplace violence on IWB, we aim to discover how the inner dimensions of workplace violence affect IWB. According to the relevant literature, we subdivide workplace violence into three specific dimensions, namely, mobbing, harassment, and sabotage [46]. Through the test of the relationship between these sub-dimensions and IWB, the disastrous consequence of workplace violence will be revealed.

#### 3.1.1. Workplace Harassment and Innovative Work Behavior

Workplace harassment and IWB are interpersonal behaviors in which specific individuals inflict intentional harm on other individuals in the workplace [14]. Various forms of sexual harassment exist in the workplace [10]. Extreme harassment may include homicide and personal attacks, but the more common forms are lewd gestures, dirty expressions, threats, shouting, silent treatment, and devaluation. The types of harassment described here are not caused by the gender or race of the victim [47]. Although sexual harassment has received widespread attention in the American literature, other forms of general workplace sexual harassment are even more common [26]. More importantly, in European studies, the reported prevalence of non-sexual harassment was significantly higher than that of unnecessary sexual attention [43]. In some western European countries, more extensive anti-harassment legislation also clarifies the legality of non-sexual psychological harassment as a form of harassment [46]. Although harassment is a kind of individual behavior, if the organization lacks the corresponding mechanism to restrain this behavior, the victims often regard the organization’s omission as acquiescence to harassment. From the COR theory perspective, when facing an organization that cannot protect its own resources from infringement, employees often take corresponding inaction measures to prevent further losses. Therefore, if the organization cannot effectively prevent workplace harassment, affected employees will protect the remaining resources by reducing the IWB initiative. Therefore, this study proposes the following.

**Hypothesis 1a (H1a).**  *Workplace harassment negatively influences IWB.*

#### 3.1.2. Workplace Mobbing and Innovative Work Behavior

Workplace mobbing can be described as a covert process in which the perpetrator acts collectively to perform psychological attacks on the target to injure and force the other party to leave [14]. Harassment is usually a one-to-one injurious behavior, whereas mobbing is a many-to-one collective aggressive behavior. Concurrently, mobbing is different from the general conflict, but with high frequency (statistical definition: at least once a week) and lasts for a long time (statistical definition: at least six months) [48]. Most workplace mobbing is downward mobbing from the superior leader of the victim. Empirical research shows that because superior leaders often have the power to determine employees’ performance evaluation and job promotion, workplace mobbing will not only cause psychological and physical harm to the victims but also bring them income reduction or job loss problems [49,50]. According to COR theory, employees find it difficult to make positive contribution behaviors, such as IWB, under the potential pressure of physical and mental injuries, financial loss, and unemployment. Therefore, this study puts forward:

**Hypothesis 1b (H1b).**  Workplace mobbing negatively influences IWB.

#### 3.1.3. Workplace Sabotage and Innovative Work Behavior

Workplace sabotage is a kind of anti-production behavior initiated by employees either individually or collectively [51,52]. This behavior usually takes three specific forms: destruction: the act of damaging and destroying the working environment; (2) in action: foreseeable damage caused by intentional omission; (3) wear and tear: the act of intentionally causing loss of raw materials [53]. Unlike harassment and mobbing, workplace sabotage is usually an action aimed at an organization, not a specific individual. However, in an organization with workplace sabotage behavior, even if it is not the target, employees will still have immense negative feelings. This is because, for employees who are conscientious in the organization, when the results of their hard work are destroyed or depleted by sabotage, they are bound to feel skeptical and disappointed about the future of the organization. According to COR theory, when employees expect the output of their own efforts to be infringed by sabotage behavior, they will reduce the positive input, thus reducing the probability of IWB. On this basis, this study puts forward:

**Hypothesis 1c (H1c).**  Workplace sabotage negatively influences IWB.

### 3.2. Mediating Effect of Wellbeing as a Non-Utilitarian Motive

Happiness is a broad concept, which leads to the complexity of the structure of employees’ Wellbeing [54]. Employee wellbeing is a connotative concept, which can express simple feelings of well-being or wellbeing, or it can be a pluralized mental structure, including an objective list, preference satisfaction, and mental state [55]. Fundamentally, employee wellbeing is simply defined as a positive emotional state generated by the evaluation of the work or work experience [27]. Some studies have suggested that it includes psychological indicators, such as emotion, anxiety and depression, including physiological indicators, such as blood pressure, a heart condition, and general physical health [56]. On the basis of the achievements of previous studies, Daniels [57] proposed five specific dimensions of happiness measurement, namely, anxiety–comfort, depression–happiness, boredom–enthusiasm, fatigue–vitality, and anger–calm, which laid the foundation for follow-up research.

Numerous studies show that workplace violence has a serious negative effect on employees’ health and employee wellbeing, organizational performance, and even the social environment [58,59]. At the individual level, WV decreed that job satisfaction affects work-related stress [60,61]. Trépanier, Fernet [62] developed a model on the basis of self-determination theory, in which violence in the workplace predicts poor mental health and employee wellbeing linked with a lack of satisfaction of basic psychological needs (autonomy, ability, and interpersonal relationships). The results imply that workplace violence (WV) reduces the performance, autonomy, ability, and interpersonal relationship of employees, but is positively related to burnout and lack of autonomy. Thus, workplace violence will bring employees anxiety, depression, fatigue, anger, and other experiences that are not conducive to employees’ wellbeing, which can likely damage employees’ well-being [63,64].

On the one hand, many studies have suggested that employee wellbeing will significantly affect employees’ job performance and organizational citizenship behavior [57,65,66]. According to COR theory, the pressure on resource or potential losses will prompt employees to try to protect existing resources and reduce investment to avoid taking further risks [4,44]. On the other hand, employee wellbeing will affect employees’ perception of resources. According to the relevant research on psychological resources, employee wellbeing itself is also a potential resource that employees can obtain from work Hobfoll [67] and, in some cases, it will become an important reason for employees to work [29]. Therefore, when employees feel that they cannot acquire the expected happiness from their existing jobs and positions, they will reduce the input of other related resources from the point of view of protecting existing psychological resources. Naturally, this will also damage their job performance and organizational citizenship behavior [44]. Given that IWB is a kind of organizational citizenship behavior that can lead to positive performance, we believe that the loss of happiness is likely to reduce employees’ IWB. In sum, we propose:

**Hypothesis 2a (H2a).**  Wellbeing mediates between *workplace* harassment and IWB;

**Hypothesis 2b (H2b).**  Wellbeing mediates between *workplace* mobbing and IWB;

**Hypothesis 2c (H2c).**  Wellbeing mediates between *workplace* sabotage and IWB.

## 4. Materials and Methods

### 4.1. Instrument Designing

In this study, we used the questionnaire survey approach [68]. We used workplace violence (harassment, mobbing, ostracism, and sabotage) as independent variables and innovative work behaviour as a dependent variable, with employee wellbeing as a mediator. In this instrument, 22 items were used with the five-point Likert-Scale (1 stands for “strongly disagree,” and 5 stands for “strongly agree). Initially, the research instrument was developed in English but was translated into Chinese for understanding, since the samples were from China. We opted for a translation and back-translation methodology to ensure the consistency of Chinese and English versions. A pilot study was conducted. The pilot study participants suggested some changes in the research instrument. Therefore, the instrument was corrected. The revised instrument was distributed for data collection.

### 4.2. Sampling and Data Collection

To test the hypotheses, on the basis of the method of Lien and Cao [69], we distributed questionnaires to entrepreneurs of SMEs located in Guangdong Province (China). We opted to conduct an online study through www.wjx.cn (an online data collection web portal) to measure the relationship between workplace violence, employee wellbeing and innovative work behavior. We distributed 550 questionnaires among the workers of SMEs entrepreneurs located in Guangdong Province, China. We received 360 research questionnaires from these questionnaires, and 24 questionnaires were uncompleted. The complete sample size was 336, which is 61% of the distributed questionnaires.

### 4.3. Variables Measurements

The purpose of this study was to identify how to work violence (harassment, mobbing, and sabotage) directly and indirectly affects workers’ innovative work behavior, with happiness as the mediating variable. On the scale of harassment, we used the research of Rasool, Maqbool [46] as a reference. Second, we used the research results of Rasool, Wang [14] as the scale of mobbing. Third, the scale of sabotage was from the study of Harris and Ogbonna [70]. Moreover, items for the mediation variable wellbeing were obtained on the basis of the scale developed by Warr [71]. Finally, we measured IWB by six items adapted from Scott and Bruce [72]. In this study, we prepared 27 items in total and adopted a five-point Likert scale (1 point means strongly disagree, and 5 points mean strongly agree). After the scale was determined, we conducted a preliminary test on its reliability and validity and revised the scale according to the suggestions of the respondents in the preliminary test. The acceptable Cronbach’s alpha value is 0.7 or higher. The specific situation of the scale is as follows:

We assessed employees’ exposure to workplace harassment with a well-adjusted, 4-item Likert scale developed by Rasool, Maqbool [46]. All the items were measured using a 5-point Likert scale. Sample items are “My supervisor/co-worker/subordinate often shares some dirty jokes with me,” and “My supervisor/co-worker/subordinate often appreciates my figure.” (Cronbach’s α = 0.875).

Workplace mobbing used four items, which was developed on the basis of Rasool, Wang [14]. The questionnaire was scored in the form of a 5-point Likert scale. Sample items are considered: “I am often given tasks with unreasonable deadlines by my supervisor/co-worker/subordinate,” and “I am often ignored or facing a hostile reaction when I approach in my workplace”. (Cronbach’s α = 0.829).

Workplace sabotage used a nine-item Likert scale developed by Harris and Ogbonna [70]. However, according to the research needs, the items of innovation work behaviour were changed into 4. The questionnaire was scored in the form of a five-point Likert scale. Sample items are “My supervisor/co-worker/subordinate here hurries me when they want to,” and “It is common practice in my workplace to ‘get back’ at employees.” (Cronbach’s α = 0.869).

Wellbeing used an adjusted four-item Likert scale developed by Warr [71]. We measured how the questionnaire was scored in the form of a five-point Likert scale. Sample items are considered: “How happy do you think you are right now,” and “How satisfied are you with your life in general?” (Cronbach’s α = 0.868).

We measured IWB by six items adopted by Scott and Bruce [72]. All six items were measured using a five-point Likert scale. Sample items are “I always search out new technologies, processes, techniques, and/or product ideas,” and “I am innovative.” (Cronbach’s α = 0.875).

### 4.4. Demographics

We distributed 550 questionnaires among the workers of SMEs entrepreneurs located in Guangdong Province, China. We received 360 questionnaires. Therefore, 24 questionnaires were uncompleted. The complete sample size was 336. The details of the demographics of this study are shown in Table 1. Moreover, Table 1 indicates that the proportion of males and females is relatively average, among which the male sample accounted for 48.8%, and the female sample accounted for 51.2%. In terms of age composition, the samples of 18–25 years old accounted for 18.8, 25.6% from 25–30 years old, 12.5% were 31–40 years old, 27.1% were 41–50 years old, and 16.1% were 51–60 years old. In terms of education level, 9.8% of the respondents had a degree below high school, 8.9% had a high school degree, 58.6% had a bachelor’s degree or junior college degree, 10.1% had a master’s degree, and 12.5% had a doctor’s degree or above. In terms of work experience, 35.4% of the samples have worked for less than five years, 8.9% have worked for 5–10 years, 12.2% have worked for 10–15 years, 28.6% have worked for 15–20 years, and 14.9% have worked for more than 20 years.

## 5. Results

In this study, to test the direct and indirect relationship, we employed structural equation modeling (SEM), using AMOS-23, to test the hypotheses. We adopted AMOS structural equation modeling instead of partial least squares structural equation modeling. The application of the SEM in this study has the following two advantages. First, the structural model is complex and contains a series of dependent relationships. Second, the attitude, behavior, and other variables cannot be simply measured with a single item, and certain errors exist. At the same time, the SEM can allow independent and dependent variables to contain measurement errors. Second, in structural equations, factor analysis of latent variables and correlation analysis of variables can be carried out simultaneously, which can effectively reduce the complexity of the analysis.

### 5.1. Discriminant Validity of Variables

To test the structural validity and discriminant validity of core variables, this study conducts confirmatory factor analysis (CFA) on workplace harassment and workplace mobbing. As shown in Table 2, compared with the other three competing models, the five-factor model has the best fitting, which indicates that the five variables in this study have good discriminant validity.

### 5.2. Descriptive Analysis and Correlation Analysis

As indicated in Table 3, workplace mobbing (r = 0.671, *p* < 0.05) and workplace sabotage (r = 0.643, *p* < 0.01) presented a significantly positive correlation with workplace harassment and negative correlation with wellbeing (r = −0.635, *p* < 0.01) and IWB (r = −0.748, *p* < 0.01). The relationship between workplace mobbing and workplace sabotage was positively correlated (r = 0.408, *p* < 0.01) and negatively correlated with wellbeing (r = −0.27, *p* < 0.01) and IWB (r = −0.25, *p* < 0.01). Workplace Sabotage was negatively correlated with wellbeing (r = −0.523, *p* < 0.01) and IWB (r = −0.583, *p* < 0.01). A significant positive correlation existed between wellbeing and IWB (r = −0.523, *p* < 0.01).

### 5.3. Direct Effects

In this study, we used the structural equation model of AMOS-23 (IBM, Armonk, NY, USA) to test the direct effects. Table 4 shows the significance of the direct effect and its relationship in the theoretical framework. The results of this study are as follows. First, WH negatively influences IWB (β = −0.799; *p* < 0.000), which confirmed the H1a. This result implies that once workplace harassment activities increase within the organization, the IWB of the staff will decrease. Second, workplace mobbing also negatively influences the IWB of the staffs (β = −0.860; *p* < 0.000), which confirmed the H1b. That is to say, if workplace mobbing increases, then IWB will decrease. Third, workplace sabotage, as an independent variable, has a negative relationship with IWB as well (β = −0.648; *p* < 0.000), thus H1c is also accepted. Table 4 presents the details related to these hypothesis testing.

### 5.4. Indirect Relationship Testing

Similar to the direct effects, we used the SEM analysis of AMOS-23 to test the mediating effects in the study hypothesis. Table 5 depicts that employee wellbeing is mediated by the relationship between workplace violence and IWB. The results show that WH negatively affects employees’ wellbeing (β = −0.710; *p* < 0.000), and staff wellbeing positively impacts their IWB (β = 0.770; *p* < 0.000). Moreover, workplace harassment is also negatively correlated with IWB (β = −0.263; *p* < 0.000). A significant mediating effect exists between workplace harassment and IWB. Similarly, in H2b, we examined the mediating effect of employee wellbeing on the relationship between workplace mobbing and IWB. The results show that a negative correlation exists between workplace mobbing and employee wellbeing (β = −0.820; *p* < 0.000), while wellbeing has a positive impact on their IWB (β = 0.903; *p* < 0.000), and a negative correlation also exists between workplace mobbing and IWB (β = −0.140; *p* < 0.015). That is, the mediating effect of employee wellbeing between workplace mobbing and IWB is significant. Similarly, in H2c, we test the mediating effect of employee wellbeing on workplace sabotage and IWB. Resultantly, workplace sabotage negatively affects staffs’ wellbeing (β = −0.613; *p* < 0.000), and the employee wellbeing of employees is positively correlated with their IWB (β = 0.915; *p* < 0.000), while workplace sabotage negatively impacts IWB as well (β = −0.099; *p* < 0.019). That is to say, the mediating effect of employee wellbeing on workplace sabotage and IWB is significant. Therefore, indirect H2a, H2b, and H2c were also accepted.

## 6. Discussion

With the impetus to find the direct effects of workplace violence on innovative work behaviour, and the intervening influence of employee well-being, this research effort has provided insightful results based on the synthesized model framework. To the best of the author’s knowledge, this research is among the earliest research paradigms to investigate the impact of workplace violence on innovative work behaviour in the workers of SMEs entrepreneurs, especially by considering employee well-being as a mediating construct.

First, we focused on the direct relationship between workplace violence (workplace harassment, workplace mobbing, and workplace sabotage) and innovative work behavior. The results show that workplace violence has a negative relationship with IWB, which supports the intuition drafted in hypothesis H1a–H1c of the study. Previous studies showed that workplace violence has a negative relationship with innovative work behavior [73,74,75,76]. Rasool, Wang [14] conduct study in the Pakistani organizations, and the outcomes of their study indicate that workplace violence is directly negatively connected with sustainable work performance that effect the worker’s innovative work behavior. However, the empirical result of this study shows that workplace violence can reduce employees’ IWB. By proposing a new lens of negative constraints of external factors, this finding enriches the existing IWB analysis, thinking mainly of the basis of internal causes and positive drivers.

Second, the mediated effect of employee well-being also translates into significant results, which supports hypotheses H2a–H2c of this study. Prior studies also support the findings of our research [77,78]. Moreover, the results of Khoreva and Wechtler [79] also support the outcomes of our study. Similarly, Khoreva and Wechtler [79] conducted a study among 300 employees and 34 immediate supervisors in a professional service company in Finland. The outcomes of that study support our study and suggest that employee well-being is a significant element increasing the innovative work behavior of employees [80]. However, according to the empirical results of this study, IWB may also be an organizational citizenship behavior driven by Wellbeing. This idea is consistent with the conclusion by Sharifirad [81] that IWB can also promote employees’ wellbeing. That is, IWB is not purely a utilitarian behavior but an organizational, civic behavior that can be driven by wellbeing and also bring wellbeing. Therefore, the findings in this study enrich the relevant research of IWB from the perspective of broadening the types of motivation sources.

Furthermore, in this study, we pinpoint an important organizational risk with COR theory, namely, the degree to which the consequences of workplace violence could be underestimated. With the continuous improvement in market uncertainty and the persistent upgrade of service personalization, the survival and development of enterprises increasingly depend on grassroots employees to creatively provide personalized products and services for different customers. Therefore, the level of employees’ IWB has become a key indicator of the success or failure of today’s enterprises [40]. COR theory theorizes that human behavior is motivated by the maintenance of existing resources and the pursuit of potential resources. [67].

## 7. Conclusions, Limitations and Future Research

### 7.1. Conclusions

The empirical model of this study is based on the previous foundation and the insights provided by the COR theory. In this study, our findings confirm the linkages among WV, employee wellbeing and innovative work behavior among workers in SMEs in China. The finding of this study indicates that in the direct relationship, WV negatively influences IWB among Chinese workers in SMEs entrepreneurs. This research paper also confirms that employee wellbeing mediates the relationship between WV and IWB.

The outcomes of this research are concluded. In the direct and indirect relationship workplace harassment negatively affect the innovative work behaviour of employees, that bring negative feelings among employees. The feelings that come with workplace harassment can be detrimental and lead to unnecessary anxiety towards co-workers. Moreover, the workplace mobbing increases the level of anxiety, stress, irritability, low work engagement, absence of work performance, and work destruction. Furthermore, workplace sabotage reduces the motivation among employees and organizations, which reduced work efficiency. Similarly, employee wellbeing positively mediates between workplace violence and innovative work behaviour. This indicates that if organizations eare about their employees, in return, employees give them innovative work behaviour. On the other hand, employee wellbeing will affect employees’ perception of resources. According to the relevant research on psychological resources, employee wellbeing itself is also a potential resource that employees can obtain from work and, in some cases, it will become an important reason for employees to work [29]. The Chinese Government should introduce a policy for both physical and psychosocial work environments, which in return will reduce workplace violence and promote well-being among workers in SMEs in China. This recommendation will help to create a workers’ well-being environment among SMEs entrepreneurs.

Lastly, from the COR theory perspective, this study further emphasizes the potential risks caused by workplace violence. Previous work violence studies paid more attention to the factors that affect the short-term performance of enterprises, such as workplace violence and productivity, and seldom discussed their long-term impact on organizations. IWB is a key factor that minimally affects the organization in the short term but has a profound impact on the growth and development of the enterprise in the long term. By discussing the impact of workplace violence on IWB, this study reveals the potential long-term negative effects of workplace violence on enterprises.

### 7.2. Limitations and Future Research

A relatively small sample is one of the limitations of this study. Future research may test the proposed model with a larger and more diversified sample to further extend the validity of the results. The findings of this study only investigate SMEs entrepreneurs. Further research can be done in other sector, such as manufacturing and IT in order to generalize the results or modify the concepts. Lastly, the future study may also enlarge the present framework by merging RBV and KBV with other performance-based theories. Therefore, future research may explore the relationship between these factors and WV and workers’ productivity using organizational support or organizational culture as a mediating variable.

## Figures and Tables

**Table 1:** Demographics.

Demographic	Items	Frequency	Percentage (%)
**Gender**	Male	164	48.8
	Female	172	51.2
**Age**	18–25	63	18.8
	25–30	86	25.6
	31–40	42	12.5
	41–50	91	27.1
	51–60	54	16.1
**Education**	Under the high school	33	9.8
	High School	30	8.9
	Bachelor degree/Junior college degree	197	58.6
	Master	34	10.1
	Ph.D. and above	42	12.5
**Work Experience**	Less than 5 Years	179	35.4
	5–10 Years	30	8.9
	10–15 Years	41	12.2
	15–20 Years	96	28.6
	More than 20 Years	50	14.9

**Table 2:** Confirmatory Factor Analysis.

Model	Factors	x2	Df	x2/df	Δ2/	RMSEA	TLI	CFI	SRMR
**Model 1**	WH;WM;WS;WB;IWB	455.74	199	2.29	-	0.06	0.94	0.95	0.04
**Model 2**	WH + WM + WS;WB;IWB	860.987	206	4.18	405.25 **	0.1	0.84	0.86	0.07
**Model 3**	WH + WM + WS + WB;IWB	1193.264	208	5.74	737.52 **	0.12	0.77	0.79	0.08
**Model 4**	WH + WM + WS + WB + IWB	1207.245	209	5.78	751.51 **	0.12	0.76	0.79	0.08

Note: Root Mean Square Error of Approximation = RMSEA, Tucker-Lewis index = TLI, Comparative Fit Index = CFI, Workplace Harassment = WH, Workplace Mobbing = WM, Workplace Sabotage = WS, Well-being = WB, Innovative Work Behaviour = IWB. (**) demonstrate the significance of the variables’ relations (*p* < 0.01 is considered significant).

**Table 3:** Mean, Standard Deviation, and Correlations of Variables.

Variables	M	SD	1	2	3	4	5
**1. WH**	3.445	0.920	1				
**2. WM**	2.734	0.704	0.671 **	1			
**3. WS**	3.445	0.800	0.643 **	0.408 **	1		
**3. WB**	2.546	0.833	−0.635 **	−0.566 **	−0.523 **	1	
**4. IWB**	2.619	0.750	−0.748 **	−0.624 **	−0.583 **	0.865 **	1

Note: (**) demonstrate the significance of the variables’ relations (*p* < 0.01 is considered significant). Mean = M, Standard Deviation = SD, Workplace Harassment = WH, Workplace Mobbing = WM, Workplace Sabotage = WS, Well-being = WB, Innovative Work Behaviour = IWB.

**Table 4:** Direct Effects (Path model results).

Hypothesis		Estimate	S.E.	C.R.	*p*
**H1a**	IWB ← WH	−0.799	0.064	−12.403	***
**H1b**	IWB ← WM	−0.860	0.086	−10.050	***
**H1c**	IWB ← WS	−0.648	0.064	−10.128	***

Note: (***) demonstrate the significance of the variables’ relations (*p* < 0.05 is considered significant generally). S.E., standard error; C.R., composite reliability; WH = Workplace Harassment, WM = Workplace Mobbing, WS = Workplace Sabotage, IWB = Innovative work behavior.

**Table 5:** Indirect Effects (path model results).

Hypothesis		Estimate	S.E.	C.R.	*P*
**H2a**	WB ← WH	−0.710	0.064	−11.056	***
	IWB ← WB	0.779	0.063	12.289	***
	IWB ← WH	−0.263	0.046	−5.687	***
**H2b**	WB ← WM	−0.820	0.086	−9.504	***
	IWB ← WB	0.903	0.069	12.046	***
	IWB ← WM	−0.140	0.058	−2.424	0.015
**H2c**	WB ← WS	−0.613	0.065	−9.398	***
	IWB ← WB	0.915	0.067	13.622	***
	IWB ← WS	−0.099	0.042	−2.340	0.019

Note: (***) demonstrate the significance of the variables’ relations (*p* < 0.05 is considered significant generally). S.E., standard error; C.R., composite reliability; WH = Workplace Harassment, WM = Workplace Mobbing, WS = Workplace Sabotage, WB = Wellbeing, IWB = Innovative work behavior.
